# Efficacy of microbial sampling recommendations and practices in sub-Saharan Africa

**DOI:** 10.1016/j.watres.2018.01.054

**Published:** 2018-05-01

**Authors:** David D.J. Taylor, Ranjiv Khush, Rachel Peletz, Emily Kumpel

**Affiliations:** a Department of Mechanical Engineering, Massachusetts Institute of Technology, Cambridge, MA USA; b Aquaya Institute, P.O. Box 5502, Santa Cruz, CA 95063, USA; c Aquaya Institute, Riara Corporate Suites, Suite #203, Riara Road, Kilimani Estate, Nairobi, Kenya; d Department of Civil and Environmental Engineering, University of Massachusetts Amherst, MA 01003 USA

**Keywords:** Sampling programs, Microbial water quality, Guidelines for drinking water quality, Sub-saharan Africa, Statistical uncertainty, Water quality regulations

## Abstract

Current guidelines for testing drinking water quality recommend that the sampling rate, which is the number of samples tested for fecal indicator bacteria (FIB) per year, increases as the population served by the drinking water system increases. However, in low-resource settings, prevalence of contamination tends to be higher, potentially requiring higher sampling rates and different statistical methods not addressed by current sampling recommendations. We analyzed 27,930 tests for FIB collected from 351 piped water systems in eight countries in sub-Saharan Africa to assess current sampling rates, observed contamination prevalences, and the ability of monitoring agencies to complete two common objectives of sampling programs: determine regulatory compliance and detect a change over time. Although FIB were never detected in samples from 75% of piped water systems, only 14% were sampled often enough to conclude with 90% confidence that the true contamination prevalence met an example guideline (≤5% chance of any sample positive for FIB). Similarly, after observing a ten percentage point increase in contaminated samples, 43% of PWS would still require more than a year before their monitoring agency could be confident that contamination had actually increased. We conclude that current sampling practices in these settings may provide insufficient information because they collect too few samples. We also conclude that current guidelines could be improved by specifying how to increase sampling after contamination has been detected. Our results suggest that future recommendations should explicitly consider the regulatory limit and desired confidence in results, and adapt when FIB is detected.

## Highlights

• Analyzed microbial water quality sampling practices in 351 piped systems in sub-Saharan Africa.
• Many systems detected no indicator bacteria but tested too few samples to be confident in results.
• Sampling recommendations should consider definitions, limits, and confidence levels.
• These results can be used to improve international guidelines for water quality monitoring.

## 1 Introduction

While more than 2.6 billion people have gained access to an improved water source over the last 25 years, recent evidence suggests that many of these improved sources do not provide drinking water that is safe (Onda et al., 2012, Bain et al., 2014a, Shaheed et al., 2014, WHO and UNICEF, 2015). Measurements of water quality are important for managing and controlling water safety and tracking progress to national and global targets such as the Sustainable Development Goals (WHO and UNICEF, 2014, WHO and UNICEF, 2017). Water management agencies around the world sample microbial drinking water quality to assess whether systems provide water that minimizes risks to health (Rahman et al., 2011, Peletz et al., 2016). Water quality can be monitored for regulatory or operational purposes: regulatory (or verification) monitoring is performed to ensure that a water supply meets standards, while operational monitoring is used to assess operations and detect changes in performance (WHO, 2011). However, collecting samples and testing water quality can be expensive, time-consuming, and logistically complicated (Crocker and Bartram, 2014, Wright et al., 2014, Bain et al., 2014b, Peletz et al., 2016).

Many countries in sub-Saharan Africa (SSA) have adopted or adapted the recommendations in the World Health Organization (WHO) Guidelines for Drinking Water Quality (GDWQ) for the design of their sampling program (Peletz et al., 2016). In practice, many water management agencies in SSA conduct some testing but have not tested enough samples to meet the GDWQ recommendations for the number of samples tested (Peletz et al., 2016). Therefore, optimizing testing is a priority, particularly in low-resource settings where testing activities are constrained and the prevalence of contamination tends to be higher. Previous research on improving the efficacy of sampling has focused on piped supplies in high-income countries that have generous information about their network (e.g. historical data, pipe maps), reliably continuous water supply, and infrequent contamination (Speight et al., 2004, Grayman et al., 2007, van Lieverloo et al., 2007, Horowitz, 2013, Rosen et al., 2009). However, corresponding analyses on improving the effectiveness of sampling in middle- and low-income countries, which often have limited system data, unreliable supplies, and frequent contamination, have not been performed (Lee and Schwab, 2005). To account for the higher prevalence of contamination, more frequent sampling and different statistical methods may be necessary; current sampling recommendations do not address this possibility.

Sampling plans should achieve the goals of a monitoring program while balancing accuracy of results with ease of application and understanding. The GDWQ recommend a minimum number of samples for FIB be tested annually for regulatory monitoring (WHO, 2011); the 2011 GDWQ substantially increased sampling in very large systems (where budgets and health risks may both be larger) and switched from monthly to yearly targets (which may decrease operational costs) as compared to previous editions of the GDWQ (WHO, 1993, WHO, 2008). The GDWQ also recommend that none of the tested samples should be found positive for FIB (WHO, 2011). Standards such as these which focus on the allowable number of positive samples are easy to implement and understand, but their statistical power cannot be evaluated. However, in practice, standards that allow no positive samples are indistinguishable from high percentile standards (e.g. ≤1% chance of detecting FIB in any sample), whose statistical power we can assess (Ellis, 1989). Similarly, Hunter (2002) compared assessing compliance of bathing water with regulations using a threshold approach (95% of samples complying with water quality standards) to using a percentile approach (the 95th percentile of observed values should not exceed the water quality standard) and found that the percentile method complicated the calculation without changing the regulatory decision. While sampling programs should consider resource and logistical constraints, it is also important to have confidence in the accuracy of the information obtained from a water quality monitoring program, as false positives could lead to expending resources unnecessarily, and false negatives could result in detrimental health consequences.

We analyze what can be learned from past monitoring programs that tested piped water quality in SSA by evaluating each system's: 1) sampling rate and contamination prevalence; 2) ability to assess compliance against a regulatory limit; and 3) ability to detect changes in water quality over time. Our aim is to identify how to increase the effectiveness of sampling recommendations for piped water systems in low-resource settings; we use these results to inform our recommendations for improving current sampling practices and guidelines.

## 2 Methods

### 2.1 Data collection

Water quality data were collected as part of the Monitoring for Safe Water (MfSW) program from eight countries in SSA: Benin, Ethiopia, Ghana, Guinea, Kenya, Senegal, Uganda, and Zambia (Peletz et al., 2013, Peletz et al., 2016, Kumpel et al., 2016). A full description of the MfSW program and participating agencies are described in Peletz et al. (2016) and Kumpel et al. (2016). Sampling and testing of drinking water was conducted by monitoring agencies responsible for regulatory monitoring of water quality. These included water suppliers (*Supplier*), responsible for providing and monitoring piped water, and health or water surveillance agencies (*Surveillance*), responsible for monitoring and ensuring the quality of all water sources in their jurisdiction. Some information about sampling locations were available for 71% of samples in the database, although these include varying levels of detail (Fig. S1).

Water quality data were collected during two stages of MfSW: 1) retrospective data, collected from agencies that had applied to MfSW (with samples tested between Jan 2009–Dec 2013) and 2) MfSW-supported data, collected by participating monitoring agencies every month (with samples tested between Jul 2013–Apr 2015). The average number of samples taken over time (*sampling rate*) by a given agency increased during the MfSW program since the program provided financial support and incentives to agencies to reach sampling rate targets (Peletz et al., 2013); therefore, the retrospective data represents baseline conditions while the MfSW-supported data represents a ‘best-case-scenario’ for sampling rate.

### 2.2 Statistical model for water quality in a piped water system

The microbial quality of piped water varies spatially and temporally throughout a system (Ellis, 1989, Geldreich, 1996). Spatial variations may be introduced by increasing water age, consumption of free chlorine, or point sources of contamination (e.g. backflow or intrusion). Similarly, temporal variations may be induced by changes in source water quality, treatment efficacy, system parameters (e.g. flow rates or pressures), momentary low pressure events (e.g. from maintenance or pressure transients), or time varying contaminant sources (e.g. increased intrusion after rainfall). Any water quality sample that tests positive for FIB provides the utility with specific and conclusive evidence of a problem that existed at a specific time and a specific location in their system. However, from a regulatory perspective, the important question is not the quality of a specific sample at location X and time Y, but, on average, the overall safety of the water distributed by this piped water system (Ellis, 1989). Ellis (1989) suggests four possible ways to account for the temporal and spatial variability of water quality: first, by randomizing samples over space and time; second, by selecting sampling locations representative of ‘average’ conditions; third, by selecting sampling locations representative of the ‘worst’ conditions; and finally, by modeling the sources and distribution of contamination and adjusting the sampling strategy accordingly.

Volume 3 of the GDWQ for community supplies recommends that sampling locations should be “representative of the water source, treatment plant, storage facilities, distribution network, points at which water is delivered to the consumer, and points of use” (WHO, 1997). More conservatively, the 2011 GDWQ recommend that samples should be taken at locations with the “best possible chance of detecting contamination” (WHO, 2011). In either case, however, repeated samples from fixed locations can bias the results (Cotter, 1985), and dramatically so where a sampling agency takes action to address contamination after it is detected.

Unfortunately, it was not possible to verify how sampling agencies selected sampling locations. Further, as sampling locations were not uniformly repeated or randomized, credibly accounting for the spatial variations in the samples was beyond the scope of this work. The primary drivers of temporal variations are seasonally- and weather-dependent, both of which are geographically dependent. Given the geographic dispersion in our dataset, accounting for climatic and weather phenomena was beyond the scope of this work.

Therefore, as a starting point for more refined statistical models in the future, and following with analyses conducted in high-income contexts (Ellis, 1989, Cotter, 1985), we combine temporal and spatial sources of variance. Specifically, we model sampling a piped water system (at any time or place) as a Bernoulli trial with a fixed probability of detecting or failing to detect FIB. Functionally, this is equivalent to modeling each piped water system as a unique, continuously-stirred reactor.

### 2.3 Definitions

#### 2.3.1 Piped water systems (PWS)

Networked water systems were sampled and tested by various monitoring agencies (water suppliers or surveillance agencies); the water quality results were then often reviewed and evaluated by water quality technicians, operators, or management in the monitoring agencies, regulators, benchmarking institutions, and other stakeholders (Kumpel et al., 2016).

For this analysis, we define a piped water system (PWS) as a networked water system in a village, town, or city that was tested by one monitoring agency (Supplier or Surveillance) whose data were either entirely in the retrospective database or entirely in the MfSW-supported database. This definition meets two goals: 1) to assess how much is known about the performance of individual water systems by each agency that tests them (i.e. we do not pool performance data as sampling agencies would not necessarily have access to the pooled data), and 2) to preserve the differences between the two datasets (since one represented a baseline condition and the other represented a ‘best-case scenario’). To achieve the first goal, we classified the same monitoring agency sampling multiple systems as separate PWS (e.g. one monitoring agency sampling two water systems was classified as two separate PWS). Similarly, we classified the same water system sampled by different monitoring agencies as separate PWS (e.g. the same water system sampled by two monitoring agencies was classified as two separate PWS). To achieve the second goal, if one water system had been sampled by one monitoring agency in both retrospective and MfSW-supported datasets, we classified it as two separate PWS in the analysis. Finally, while we use these data to illustrate the value of data for regulatory and operational scenarios, the agencies whose data we analyze were selected non-randomly; therefore, we caution against using the data to interpret the water quality of particular countries (Peletz et al., 2016).

#### 2.3.2 Contamination prevalence

Monitoring agencies reported the results of individual water quality samples tested from PWS, including the date of sample, location (e.g. town/city or sub-district), and test results. We only included data from sampling points representative of water used for drinking from a piped system (i.e. consumer taps and standpipes). We excluded samples from non-piped sources, and further upstream in a piped distribution system (e.g. raw (untreated) water, water treatment plants, and service reservoirs). We categorized a result as a test for fecal indicator bacteria (FIB) if the sample was tested for *E. coli*, fecal coliform, *Enterococci*, or H_2_S, or if agencies tested for total coliform and included a confirmation step (details in Kumpel et al. (2016)). We define a positive test based on whether a 100 mL sample was positive for FIB through a presence/absence test or quantitative method (e.g. colony forming units (CFU) or most probable number (MPN)). We excluded samples from analysis if the method systematically tested a volume less than 100 mL. We refer to the percentage of all samples taken from a PWS that tested positive for fecal indicator bacteria (FIB) as the *observed contamination prevalence*. Specifically, due to variance in the number of samples and the spacing of samples, the observed contamination prevalence does not consider the time intervals between samples. Even if nothing about the water quality in a system changes, the observed contamination prevalence can fluctuate due to chance (e.g. from one year to the next).

In keeping with our statistical model, we also consider the *true contamination prevalence* — the probability that any sample from a PWS would be positive for FIB. Conceptually, this can be thought of as the contamination prevalence that would be observed if the PWS was randomly sampled a very large number of times. The true contamination prevalence of a PWS describes the underlying state of the system and does not depend on where or how the system is sampled. It only changes when the water quality in the PWS changes. Therefore, much of the statistics that follow analyze the difference between the *observed* and the *true* contamination prevalences.

#### 2.3.3 Sampling rates

For each PWS, we calculated the target number of samples tested per year (*sampling rate*) for the system as recommended by the WHO GDWQ (*recommended sampling rate*) and compared this with the number of samples we observe recorded per year in the dataset (*observed sampling rate*). For most PWS, we had less than one year of data; while our observed sampling rates adjusted for this limited observation window, our calculations of the statistical uncertainty about a PWS' contamination prevalence used all available data (i.e. did not adjust for the observation window). For PWS with more than a year of data, this overestimated the certainty available on an annual basis; conversely, for PWS who submitted only a portion of their annual data, this underestimated the available certainty about their contamination prevalence. In either case, by using all available data, our method reflects how much regulators could learn about the contamination prevalence in each PWS from our dataset.

*Recommended:* For each town or city in the dataset, we calculated the recommended (minimum) annual sampling rate according to the WHO GDWQ: 1) 12 samples for systems serving <5,000 people; 2) 12 samples for every 5,000 people for small systems serving 5,000–100,000 people; 3) 12 samples for every 10,000 people plus 120 samples for systems serving 100,000–500,000 people; and 4) 12 samples for every 50,000 people plus 600 samples for very large systems (>500,000 people) (WHO, 2011). These sampling rates refer to the number of samples to be tested per year. For towns and cities in Ethiopia, Benin, and Zambia, we obtained population data from reports submitted through MfSW (Peletz et al., 2016). For towns and cities in Kenya, we obtained population data from the Water and Sanitation Regulatory Authority (WASREB) reports (WASREB, 2012). For towns and cities located in Uganda, we used the population listed for the town council, sub-county, or district capital from the 2014 housing census data or, for those municipalities spread over several administrative units, we summed the populations of the relevant administrative units (Ugandan Bureau of Statistics, 2014). For very small community (parish) systems in Uganda we used the mean parish size for that district or, when missing, a neighboring district, from the 2002 housing census (Uganda Bureau of Statistics, 2006). Since most parishes had populations of ≤ 5000 people (which results in a constant recommendation of 12 samples per year), the GDWQ-recommended sampling rates did not depend on exact parish populations. We were unable to obtain relevant population data for the towns and cities in the dataset from Ghana, Guinea, or Senegal, and therefore PWS from these three countries are not included in the analysis presented in Section 3.1 which compares the recommended sampling rate to the observed sampling rate. Often, countries have national standards which require a higher sampling rate than the WHO GDWQ (Peletz et al., 2016); however, here we consider only the GDWQ sampling rate for uniformity.

*Observed:* We calculated the observed annual sampling rate based on the obtained water quality data. Due to differences in incentives and program structure, we accounted for missing data differently in the retrospective and MfSW-supported data. Using retrospective data from samples tested from 2011 to 2013, we calculated the mean monthly sampling rate between the first and the last month of all months for which test results were recorded for each PWS (since fewer than 12 months of retrospective data were available for many PWS). This method inflated the sampling rate of PWS in the retrospective database that were sampled for only a few consecutive months. Using MfSW-supported data, we calculated the mean monthly sampling rate from the first year of MfSW data (during the first year, all participating PWS were supposed to have submitted data). We multiplied all mean monthly sampling rates by 12 to obtain annual sampling rates, and categorized sampling rates by data source (Retrospective or MfSW-supported).

### 2.4 Statistical methods

First, we aggregated contamination prevalences and observed sampling rates for each PWS to understand the range of sampling patterns and observations. We then evaluated whether the water quality results from a single PWS could be used to: 1) determine compliance of a PWS with an example regulatory limit (regulatory monitoring); and 2) detect performance degradation (operational monitoring). To improve existing recommendations, we demonstrated how regulatory limits and sampling rates could be harmonized and quantified how much additional sampling should be required when a given number of samples test positive for FIB.

*Determining compliance with an example regulatory limit:* The 2011 WHO GDWQ recommend no samples should be positive for FIB. We analyze only the stated recommendations and explore the statistical power and limitations of sampling programs that would follow the GDWQ. Under the current GDWQ, it is easy to determine whether or not a PWS has passed the standard; however, how confident should a monitoring agency be that the PWS in question has a true contamination prevalence of, for example, ≤5%? To facilitate this type of uncertainty analysis, we follow Ellis (1989) and convert the GDWQ into a percentile standard. The exact percentile equivalent depends on the number of samples and desired confidence level. As a starting point, we consider a limit of 5%, similar to the limit in the US 1989 Total Coliform Rule for systems testing more than 40 samples in a month. Specifically, we consider two alternative regulatory limits: in every PWS, there should be a ≤5% chance of detecting FIB in any sample with either 75% or 90% confidence. These alternative regulatory limits allow us to quantify the uncertainty caused by existing sampling practices and recommended sampling rates.

**Fig. 1:**
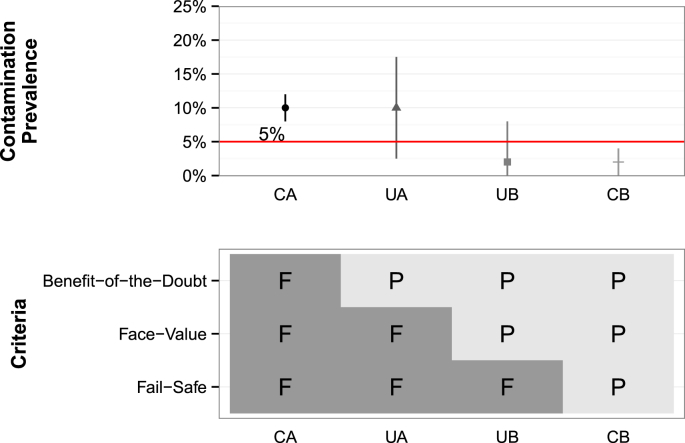
Classifying uncertainty about true the contamination prevalence and its relationship to the criteria for passing a regulatory limit of ≤5%. CA: confidently above; UA: unconfidently above; UB: unconfidently below; CB: confidently below. Each category fails (F) or passes (P) the limit as shown in the lower grid.

Since we classified samples tested for FIB as positive or negative (contained FIB or did not contain FIB in a 100 mL sample), the results were binomially distributed. Using the observed contamination prevalence, for each PWS, we evaluated the range in which the true contamination prevalence was expected to lie below 90% of the time and the range in which the true contamination prevalence was expected lie above 90% of the time (displayed as error bars), implemented using the Clopper-Pearson algorithm in R (R Core Team 2016, version 3.2.1). We then used these confidence intervals to classify all PWS in the dataset into four categories: a) confidently (with 90% confidence) above (CA) the regulated limit (5%); b) unconfidently above the limit (UA); c) unconfidently below the limit (UB); and d) confidently below the limit (CB) (Fig. 1).

Three evaluation criteria can be used to convert the four classifications into a regulatory verdict of passing or failing: *benefit-of-the-doubt*, *face-value*, or *fail-safe* (Ellis, 1989, McBride, 2005). The most relaxed is the *benefit-of-the-doubt* criterion, where the true contamination prevalence is assumed to pass the regulated limit unless proved otherwise with the required level of confidence (e.g. passes unless we have 90% confidence the true contamination prevalence is >5%; i.e. only CA fails). The most common, and current GDWQ approach, is the *face-value* criterion where the observed contamination prevalence is assumed to be the true contamination prevalence and confidence levels are ignored (i.e. CA and UA fail the standard). Finally, the most conservative is the *fail-safe* criterion, where PWS are assumed to fail the regulated limit unless proved otherwise with the required level of confidence (e.g. fails unless we have 90% confidence the true contamination prevalence is ≤5%; i.e. only CB passes). The relationship between PWS categorization and evaluation criterion is detailed in Fig. 1.

*Detecting quality degradation:* While the WHO GDWQ are designed for regulatory monitoring, results are also likely to be used by water system operators to make operational decisions and regulators to detect temporal trends. We therefore explore how much should be learned from existing sampling practices about changes in water quality. As the sampling requirements for operational monitoring are higher, we adopt a *benefit-of-the-doubt* approach — determining how long it would take before an operator or regulator could be sure that their PWS' true contamination prevalence had increased (i.e. water quality has degraded); we do not, however, quantify the probability of failing to detect a genuine change (i.e. type II error, see for e.g. McBride (2005)).

Our statistical model treats temporal and spatial variations equivalently; therefore, this section considers how many samples are required to distinguish between sample sets with observed contamination prevalences that differ. For a sampling agency with fixed sampling locations, the spatial variation is controlled for, and different contamination prevalences could be interpreted as a degradation in water quality over time. For an agency with randomized sampling locations, the difference in observed contamination prevalences could be induced by temporal or spatial variations; nevertheless, if each set of samples was taken at randomized locations and at randomized times, the difference could still be interpreted as temporally-induced with the statistical confidence suggested by the method that follows (Cotter, 1985).

We calculated the minimum number of samples required before a large (10 percentage-point) increase in the observed contamination prevalence of a PWS would lead to the conclusion that the PWS had actually experienced a degradation in water quality with a given level of statistical confidence. To achieve this, we modeled each PWS as having two sets of samples of equal size, each with an unknown true contamination prevalence. The first set was taken to have an observed contamination prevalence equal to the one in the dataset. The second set was simulated to have an observed contamination prevalence 10 percentage points higher (e.g. from 1% of samples contaminated to 11%, or from 85% to 95%). The prior assumption about the true contamination prevalence in each set of samples was independent and uniformly distributed from 0% to 100%. We used Bayesian updating to model how more samples increased the confidence that the observed change was also a true change. The specific equations used to calculate the minimum number of required samples, using Beta distributions and solved numerically, are in Text S1. The calculated minimum number of samples was converted into a minimum time by dividing the minimum number of samples by the observed sampling rate for each PWS.

*Strategies for improving sampling recommendations:* To illustrate how to align sampling recommendations and regulatory limits, we calculated the minimum number of samples required to pass a *fail-safe* regulatory limit, the most conservative definition (Fig. 1), with a given confidence, again using the Clopper-Pearson algorithm. Depending on the time horizon of study (monthly, quarterly, yearly, etc.), the minimum number of required samples can be converted into a minimum sampling rate by dividing the required number of samples by the time horizon in which data would be assessed (e.g. if 24 samples are required, and we want to assess the water quality in every quarter, this translates into 96 (=24/0.25) samples per year).

The GDWQ recommend that no samples be found positive for FIB; however, should a PWS be penalized if it is sampled 2.5 times more often than the recommended minimum rate and one sample is found to be positive for FIB? Similarly, in a PWS in which only a few samples test positive for FIB, these few samples may not be representative of their PWS′ true contamination prevalence. We therefore calculated how many more samples would need to be tested after a contaminated sample was detected to return to the original level of confidence in a PWS' true contamination prevalence. To do so, we first quantified the existing confidence in the true contamination prevalence associated with a PWS sampled according to the WHO GDWQ and with no samples found positive for FIB. We then calculated the number of additional samples that must be tested to retrieve the original level of certainty, after a given number of samples are found to be positive for FIB. This calculation was done by increasing the number of samples taken until the upper limit of the new confidence interval was equal or less than the limit without any observed contamination. For computational efficiency with large sampling numbers, we tested the nearest integer to $e^{0.01x}|x\in Z^{+}$.

## 3 Results and discussion

### 3.1 Sampling rates and observed contamination

The dataset for analysis included 351 PWS, representing 270 different distinct areas, towns, or cities in eight countries. Of these PWS, 58% were sampled by surveillance agencies and the remainder by water suppliers (Table 1). However, suppliers took more samples: 83% of samples in the dataset were tested by suppliers compared to 17% by surveillance agencies (n = 27,930). Each PWS was tested for FIB a mean of 80 times (range: 1–7765) over a mean of 9.6 months (range: 1–26) (Table 1). The mean percent of samples positive for FIB (*observed contamination prevalence*) per PWS was 11.5% (range: 0–100%) (Table 1). The breakdown of PWS sizes by country is included as Table S1. Annual sampling rates per PWS ranged from no samples per year to 7168 samples per year, with a mean of 76 and a median of 12 (Table S2). Within the subset of samples with location information (71%, n = 27,930), 47% (n = 349) of PWS were repeatedly sampled a mean of two or more times per sampling location or neighborhood (Fig. S1).

**Table 1:** Summary of the dataset by country. This summary includes descriptive statistics on the number of areas/towns/cities, samples, time period, agencies testing, and mean contamination.

Country	Areas^a^	PWS^b^	Surveillance Agencies^c^	Samples^d^	Surveillance Samples^e^	Samples per PWS^f^	Months per PWS^g^	Contamination per PWS^h^
	n	n	(%)	n	(%)	mean n (range)	mean n (range)	mean % (range)
Benin	11	11	0%	1452	0%	132 (2–1109)	4.3 (1–19)	3% (0–33%)
Ethiopia	3	4	0%	8295	0%	2074 (21–7765)	13.5 (12–17)	4% (1–10%)
Ghana	3	3	0%	506	0%	169 (37–306)	9.0 (2–13)	0% (0-0%)
Guinea	34	39	0%	2785	0%	71 (1–1494)	5.4 (1–12)	28% (0–100%)
Kenya	5	9	33%	4366	0.16%	485 (2–3491)	11.6 (1–22)	12% (0–50%)
Senegal	42	43	100%	240	100%	6 (1–61)	3.3 (1–12)	45% (0–100%)
Uganda	130	195	72%	6278	44%	32 (1–454)	12.0 (1–26)	1% (0–100%)
Zambia	42	47	40%	4008	44%	85 (1–1516)	9.4 (1–17)	13% (0–100%)

Total	270	351	58%	27930	17%	80 (1–7765)	9.6 (1–26)	11.5% (0–100%)

a Number of geographically-distinct water systems in the dataset.

b Number of piped water systems (PWS) treated separately in the analysis.

c Percent of PWS that were tested by surveillance agencies.

d Total number of samples.

e Percent of samples gathered by surveillance agencies.

f Mean number of samples per PWS.

g Mean number of months of data per PWS.

h Mean contamination prevalence (percent of samples ≥ 1 fecal indicator bacteria (FIB)/100 mL per PWS).

Three country-specific observations must be noted. First, Senegal had the highest percent of samples positive for FIB in our database at 45% (Table 1); however, data from Senegal were gathered entirely by a surveillance agency, which was more likely to test in low-resourced and rural water supplies, which are more likely to be contaminated (Peletz et al., 2016, Kumpel et al., 2016). Second, FIB were not detected in any samples from any PWS in our database from Ghana (although total coliform, not reported as an FIB, were occasionally detected), likely because only three towns were monitored, fewer samples were taken relative to other countries, and half of samples had detectable free chlorine residual. Third, PWS from Uganda are heavily represented, comprising 56% of the PWS in the dataset. We performed all analyses both with and without data from Uganda; where important, the results of this sensitivity study are noted.

**Fig. 2:**
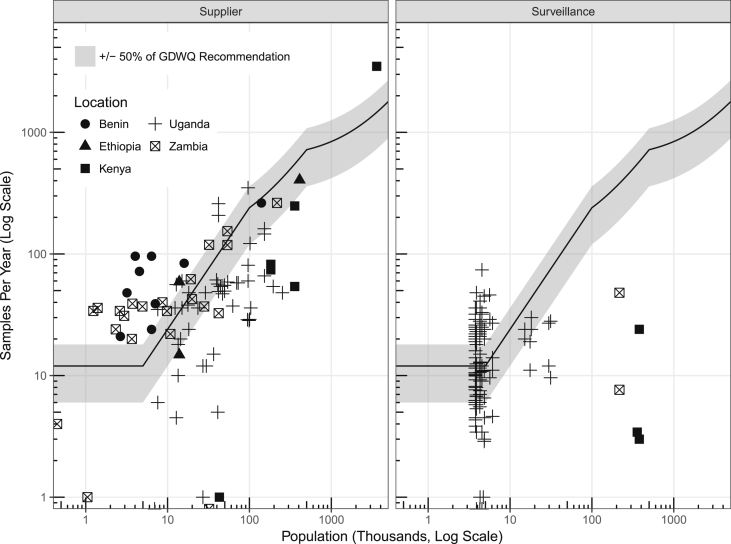
Annual equivalent sampling rates by PWS (points) compared with GDWQ recommendations (black line) and ± 50% of GDWQ recommendations (shaded grey) as tested by a) water suppliers (left) and b) surveillance agencies (right) by country (Benin (dots), Ethiopia (triangles), Kenya (squares), Uganda (crosses) and Zambia (crossed boxes)).

We compared the observed sampling rates per PWS to the recommended sampling rates calculated from the GDWQ (Fig. 2). More PWS serving smaller populations met the GDWQ recommendations than PWS serving larger populations: the median sampling rate for PWS serving fewer than 10,000 people was 108% of the GDWQ recommendation and dropped to 45% for PWS serving more than 10,000 people (Fig. 2). While supplier sampling rates generally increased with the size of the population served, surveillance sampling rates showed no increasing trend with population served: four of the five (80%) surveillance agencies monitoring PWS that served more than 100,000 people had observed sampling rates lower than 5% of the GDWQ recommended rate. While surveillance agencies are often responsible for testing every water source in their jurisdiction (e.g. piped systems, wells, springs), in practice, they often prioritize testing rural sources over urban piped sources (Peletz et al., 2016, Kumpel et al., 2016). Guidance on sampling rates for surveillance agencies is especially important when they are responsible for verifying the results of a supplier's testing; for example, the time and resources invested in surveillance agencies testing only a few samples from PWS in large cities could instead verify lab or sample collection procedures rather than conducting independent monitoring.

Sampling rates in our dataset varied substantially between countries. For example, in Kenya, only the largest supplier in the dataset sampled as frequently as the GDWQ recommendation (Fig. 2). Surveillance agencies in Kenya did not meet recommendations: they sampled PWS serving more than 100,000 people a mean of only ten times per year (the recommended rate is 240 samples per year for systems serving 100,000 people). Ugandan surveillance agencies often had a sampling rate greater than recommended in the GDWQ for small PWS, sometimes by a factor of more than six (Fig. 2). Notably, the surveillance agencies in Uganda included a regional agency with a centralized facility responsible for testing water quality for all PWS who were subscribed to the service (in place of operational monitoring conducted by the suppliers). This agency had higher institutionally-set targets than the GDWQ. Some monitoring agencies were following national sampling guidelines rather than GDWQ recommendations. Also, notably, the data collected from PWS within a country are not necessarily representative of the country-wide situation, as their selection through the MfSW program was non-random (Peletz et al., 2016).

Finally, the MfSW program was designed to increase sampling rates and succeeded. PWS included in the MfSW database were sampled at a median of 167% of GDWQ recommended rate compared to 67% of the GDWQ recommended rate in the retrospective database (Fig. S2). Therefore, the conclusions drawn from the MfSW database represent a ‘best-case-scenario.’ Of the 59 PWS that appeared in both databases, 52 (91%) were located in Uganda, where the median sampling rate increased from 64% to 175% of the GDQW recommended rate.

### 3.2 Determining compliance with an example regulatory limit

**Fig. 3:**
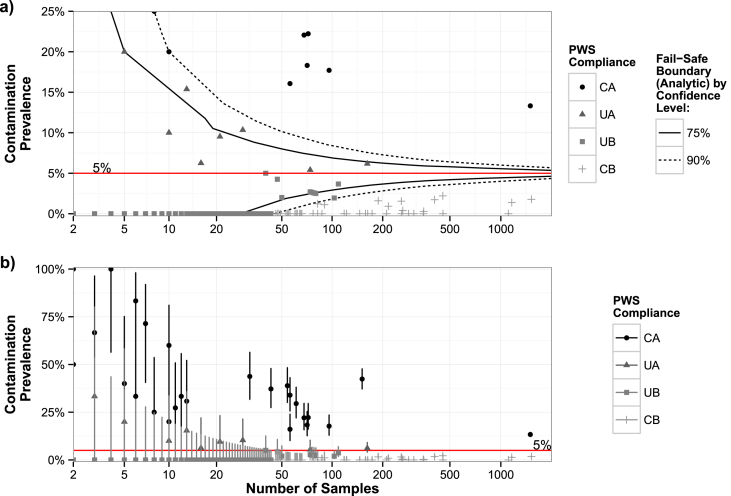
Compliance of all PWS (points) in the dataset with an example regulatory limit specifying at most a 5% chance of samples containing FIB (red horizontal line). a) Required sampling rates to pass or fail the example limit at 75% (solid lines) and 90% (dashed lines) confidence. Y-axis ranges from 0 to 25%. b) The observed contamination prevalences for all 351 PWS in the dataset with error bars that span the range of values that the observed prevalence is above with 90% confidence and below with 90% confidence. Y-axis ranges from 0 to 100%. PWS are divided into four categories according to their adherence to the limit with 90% confidence: confidently above (more contaminated than) the limit (CA), confidently below the limit (CB), unconfidently above the limit (UA), and unconfidently below the limit (UB). (For interpretation of the references to colour in this figure legend, the reader is referred to the Web version of this article.)

We simulated the minimum number of samples required to pass the example limit, given any possible observed contamination prevalence for up to 2000 samples. These simulated values were turned into the curves displayed in Fig. 3a using a convex hull algorithm.

As the observed contamination prevalence of a PWS approaches a regulatory limit (e.g. ≤5% chance of any sample being positive for FIB), more samples are required to differentiate the true contamination prevalence from the regulatory limit. However, additional samples have diminishing returns (Fig. 3a). For example, with 90% confidence, PWS that observe zero, one, or two samples positive for FIB have true contamination prevalences below the regulated limit if they sample at least 48, 105, and 900 times, respectively (Fig. 3a). PWS from our dataset are superimposed as points on Fig. 3a.

**Fig. 4:**
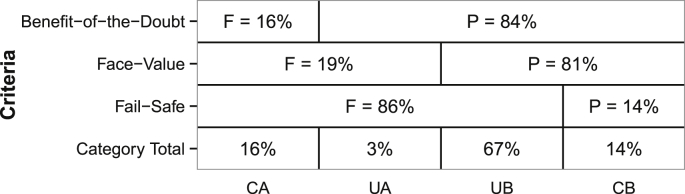
The proportion of PWS in the dataset (n = 351) that fall into each category of regulatory compliance: CA: confidently above; UA: unconfidently above; UB: unconfidently below; CB: confidently below. These categories map into failing (F) or passing (P) the regulatory limit differently for each of three criteria: benefit-of-the-doubt; face-value; and fail-safe.

Of the PWS in the dataset, only 16% (n = 351) were confidently above (CA) the example regulatory limit (i.e. with 90% confidence, the PWS′ true contamination prevalence was more than the regulatory limit) (Fig. 3, Fig. 4; each point represents data from a PWS). Conversely, only 14% were confidently below the limit (i.e. with 90% confidence, the PWS' true contamination prevalence was below the regulatory limit). For the majority of PWS, it was statistically uncertain if they were above or below the limit: 67% were unconfidently below the limit and 3% were unconfidently above (UA) the limit (Fig. 3, Fig. 4).

A *benefit-of-the-doubt* instead of a *fail-safe* criterion would shift 70% of PWS from failing to passing the regulated limit (Fig. 4). As such, providing explicit guidance on the criterion to use when applying a regulatory limit should be a feature, at least at the development stage, of future recommendations.

Using a *face-value* criterion for compliance with a limit, as the GDWQ do, which does not differentiate between observed and true contamination prevalence, has two drawbacks. First, PWS that are unconfidently below (UB) the limit will periodically fail the regulation without experiencing a change in their system. For example, a PWS sampled 24 times per year with a true contamination prevalence of 4% will fail a yearly limit of 5% an average of one quarter (25%) of the time. Second, PWS unconfidently above the limit will periodically pass the regulatory limit despite having poor water quality. For example, a PWS sampled 24 times per year with a true contamination prevalence of 8% will pass a yearly limit of 5% an average of more than a third (42%) of the time. Regulators, policy makers, and monitoring agencies must consider the uncertainty associated with sampling to avoid incorrect decisions.

The *benefit-of-the-doubt* criterion can only be justified when almost all PWS pass the regulated limit. Our dataset has a mean observed contamination prevalence of 11.5% (Table 1), and 19% of PWS have an observed contamination prevalence that is above our example limit. Therefore, we conclude that the *fail-safe* method — requiring PWS to be confident that their true contamination prevalence is ≤5% — is necessary, despite its taxing sampling requirements.

Using a *fail-safe* criterion, even though 263 of the 351 PWS (75%) had no samples containing any FIB (Fig. 3b), 227 of these were not sampled often enough to be below the limit with 90% confidence. These 227 PWS make up 65% of the total sample; this uncertainty may suggest that regulators and monitoring agencies interpret too much from a small number of samples free of FIB. Under-sampling was an even larger problem for surveillance agencies; 170 of 205 surveillance agencies (83%) never detected FIB, but 164 (97% of surveillance agencies who did not detect FIB) could not confidently conclude that the tested PWS met the regulatory limit.

To make using a *fail-safe* criterion feasible, we consider other potential regulatory limits: with current sampling practices, only half of PWS have a true contamination prevalence less than 25% with 90% confidence. Outside of Uganda, half of all PWS without observations of contamination had been sampled less than four times; such sampling programs can only conclude that contamination prevalences are less than 68% with 90% confidence. Given the expense required for testing for FIB, regulators must seriously consider if the operational information gained from knowing if a PWS' contamination prevalence is less than 25% (or 68%) justifies current expenditures. Without such considerations, current sampling practices create a false sense of knowledge and may not be an effective use of resources.

Like Hunter (2002), our data suggest the difference between *face-value* and *benefit-of-the-doubt* criteria is small in practice: only 3% of PWS in our dataset that pass a *benefit-of-the-doubt* criterion fail a *face-value* criterion. Since a *face-value* criterion is much easier to understand and apply, its use would be justified if PWS in SSA could be assumed to be in compliance with the regulated limit; unfortunately, our data do not allow for this conclusion in aggregate. Ellis (1989) suggests a useful alternative: where a *fail-safe* criterion is required, regulators may choose a much lower regulatory limit to implement with a *face-value* criterion, recognizing that if designed correctly, PWS that meet the lower limit at *face-value* will also comply with the desired (and higher) *fail-safe* limit. For example, any PWS passing a *face-value* criterion of none of ≥36 samples test positive for FIB, would also pass a *fail-safe* criterion requiring 90% confidence that the true contamination prevalence was ≤10%.

These results suggest several opportunities to improve the current testing regime. First, using higher regulatory limits (e.g. true contamination prevalence ≤10%) would decrease the number of samples required to determine compliance. Second, countries or regions with historic data that consistently passes regulatory limits may be justified in using a *benefit-of-the-doubt* criterion for evaluating their PWS. Finally, PWS sampled fewer than five times cannot pass a *fail-safe* limit of ≤50% contamination; therefore, the cost-effectiveness of dedicating resources to infrequent testing may need to be reconsidered or testing activities should be increased.

### 3.3 Detecting quality degradation

**Fig. 5:**
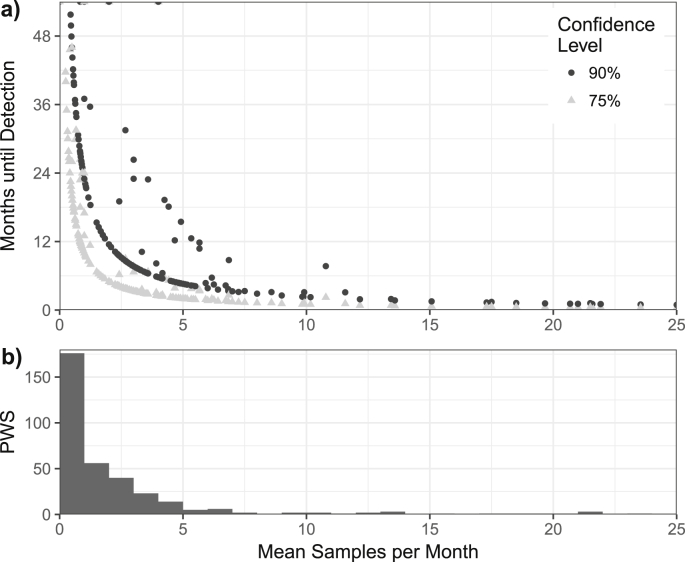
Sampling rate and ability to detect a change in water quality. a) Months until a decrease in water quality in a given PWS in the dataset could be concluded from a ten percentage point increase in the prevalence of FIB contamination with either 75% or 90% confidence. For each confidence level, the observable curve is created by PWS with extreme contamination prevalences (i.e. close to 0% or 100%). b) Distribution of the mean observed monthly sampling rates among all PWS.

From a regulatory, operational, and/or managerial perspective, it may be useful to know if a PWS′ spatially-averaged water quality is improving or degrading. Accordingly, we analyzed sampling rates and observed contamination prevalences from PWS in the dataset to understand how long it would take to conclude that a given PWS' quality had degraded once its observed contamination prevalence changed. The detection time depends on the baseline observed contamination prevalence, the magnitude of the change in observed contamination prevalence, the sampling rate, and the required confidence that the true contamination prevalence had increased. Given the observed sampling rate of a PWS, the required time before detection could occur follows a 1/x trend for each confidence level (Fig. 5a). Detecting changes in performance with less confidence (e.g. 75% instead of 90%) leads to faster detection times but more false alarms; depending on the costs of false alarms, a different level of confidence may be appropriate for different systems.

In total, with 90% confidence, we could conclude that quality had degraded for only 229 of 351 (65%) PWS within two years, 151 (43%) within a year, and only 36 (10%) within three months. For the majority of PWS in the dataset who were sampled fewer than twice per month (57%, 201 of 351) (Fig. 5b), it would take a minimum of 5 months (and a median of 13 months) to conclude that their contamination had increased, even with only 75% confidence (Fig. 5a).

If the objective of recommendations were to allow a response to changes in the observed contamination prevalence within a fixed amount of time, PWS with contamination prevalences closer to 50% would need to be sampled more often than those closer to 0% or 100% (Fig. S3). PWS with contamination prevalences close to 0% and 100% require the shortest detection times and therefore form the observable 1/x trends (Fig. 5a); as the contamination prevalence of a PWS approaches 50% the detection time deviates above the 1/x trends.

The most common sampling rate of PWS in the dataset was less than once per month (Fig. 5b), which is less than the WHO GDWQ recommends for a PWS that serves 5000. After observing an increase in contamination prevalence by 10 percentage points in one of these PWS, 23 months of data is required to conclude with 90% confidence that contamination had actually increased (Fig. 5a). A 23-month delay associated with the GDWQ recommendations for a population of 5000 (12 samples per year) would correspond to a minimum of 115,000 people-months of consumed contaminated water.

For the majority of PWS in the dataset, regulators and monitoring agencies will require more than a year to conclude with reasonable confidence (90%) that a PWS' performance has degraded after observing an increase in sample contamination by ten percentage points. Where sampling for FIB is being used as a method of detecting changes in water quality (improvement or degradation), sampling rates need to be increased or attention needs to be focused on sampling other parameters more often (e.g. operational monitoring of disinfectant residuals).

### 3.4 Strategies for improving sampling recommendations

We identified two immediate opportunities for improving regulatory sampling recommendations: 1) the minimum sampling rate and regulatory limit should be harmonized; and 2) explicit guidelines should be given for sampling PWS when FIB is detected during regulatory sampling.

Assuming that regulatory monitoring uses a fail-safe criterion, as we showed is important for this dataset, guidelines for the minimum number of samples should either be increased or the regulated limit should be increased. While the current WHO GDWQ recommend a regulated limit of 0%, this can give a false sense of security, is difficult to analyze, and can disincentivize increasing sampling. Therefore, we recommend setting a realistic and measurable regulatory limit, potentially starting with a higher limit that can be realistically evaluated based on current sampling practices.

**Fig. 6:**
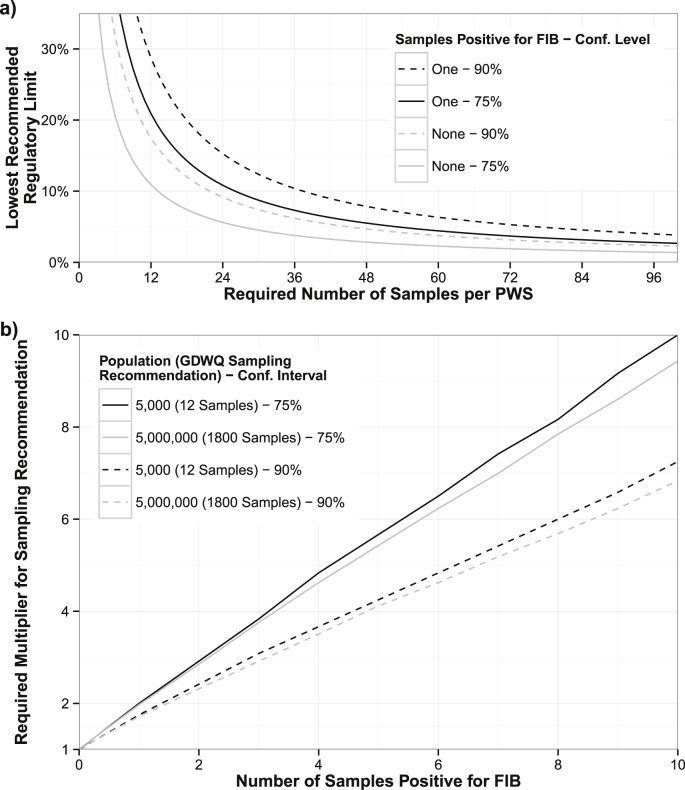
Statistical relationships for improving sampling recommendations. a) Harmonizing the number of samples and the regulatory limit for PWS with no (grey/lower lines) or one (black/upper lines) samples positive for FIB and with 75% (solid lines) or 90% (dashed lines) confidence. b) Increases in GDWQ recommended sampling rates required due to a fixed number of samples found to be positive for FIB, calculated for two population extremes: 5000 people (black/highest lines in each pair) and 5,000,000 people (grey/lower lines in each pair). The multiplier depends on the required confidence level: 75% (solid lines) or 90% (dashed lines).

To facilitate this adjustment, Fig. 6a illustrates the minimum number of samples required to have 90% and 75% confidence that a PWS passes a range of regulatory limits. For example, if sampling cannot be increased beyond 12 samples, and PWS are expected to observe no samples positive for FIB, then the lowest regulated limit should be 17.5% for regulators hoping to be 90% confident that PWS have passed the limit (Fig. 6a). Conversely, in scenarios with a specific regulated limit (e.g. ≤10% chance of any sample being positive for FIB with 90% confidence), then a minimum number of samples are required for systems that detect no FIB (≥22 samples for the same example, Fig. 6a). In general, finding even one sample positive for FIB increases the uncertainty about the true contamination prevalence of a PWS.

Specifying that no samples should be found positive for FIB (as in the GDWQ) incentivizes sampling at the minimum required rate (Ellis, 1989), and should not be recommended in future guidelines. However, if this recommendation is kept for its simplicity, additional guidance should be given on what to do when FIB is detected. A PWS in which only a few samples have tested positive for FIB may wish to protest that these samples were either an error in processing or were not representative of their PWS′ true contamination prevalence; additional sampling can evaluate this claim. Assuming that regulators use the fail-safe criterion for evaluating PWS compliance, two samples found positive for FIB require more than doubling the WHO GDWQ's recommended number of samples in order retrieve the original confidence in the true contamination prevalence (Fig. 6b).

Consider, as an example, a PWS serving 100,000 people, which should be sampled 240 times per year. If no samples are found positive for FIB, then the PWS′ true contamination rate is below 1.0% with 90% confidence. However, if two samples are positive for FIB, the PWS must be sampled an additional 366 samples (for a total of 562 samples, or 2.3 times the original sampling recommendation) before the PWS' true contamination prevalence could again be considered below 1.0% with 90% confidence. More generally, Fig. 6b suggests the required multiplier (on the y-axis) for an arbitrary number of samples found positive for FIB (on the x-axis).

Counter-intuitively, sampling regimes designed with lower confidence levels require higher sampling multipliers (Fig. 6b). This occurs because at lower confidence levels, the original certainty without a positive sample is higher. For example, with 12 samples free of FIB, the contamination is ≤ 17.5% with 90% confidence but ≤ 11% with 75% confidence. With one sample positive for FIB, even though the 75% confidence interval is smaller, it takes more samples to shift the interval low enough to be ≤11% than it does to shift the larger (90%) interval so that is it once again ≤17.5% (Fig. 6b).

Many high-income countries have adopted adaptive testing guidelines, where finding a positive sample results in a requirement for additional sampling (EPA, 2016, Commonwealth of Australia, 2011). The current version of the GDWQ does not include a similar recommendation. However, in previous work, we observed that the water suppliers did re-test sampling locations after observing contamination to verify and correct the water quality problem (Kumpel et al., 2016). Guidelines from some high-income countries and the GDWQ both provide some guidance on sampling locations, such as specifying that at least half of samples must be from the “outermost limits of the distribution system” (as in Quebec, Canada) (Cook et al., 2013, WHO, 2011); nevertheless, more detailed recommendations about sample stratification would strengthen sampling programs.

If explicit goals are given for detection limits and confidence levels, it is then possible to use these data to optimize sampling rates and how they must adapt under different contamination prevalences.

## 4 Conclusion

We demonstrated how sampling rates, contamination prevalences, desired confidence, and evaluation criterion can affect interpretation of water quality testing results. We found that:

• Most PWS in SSA in our dataset were sampled too infrequently to ensure they were providing water quality that was safely below an example regulatory limit or to make timely decisions when there was a change.
• While most guidelines rely on sampling rates based on population sizes, we demonstrated that contamination prevalences, desired confidence in results, regulatory limits, and the criterion used to evaluate results also dramatically change the usefulness of sampling programs. For example, there could be adaptive requirements wherein a PWS with a moderate number of samples positive for FIB would need to be sampled more often to compensate for the increased variance while, conversely, sampling could be reduced in a PWS with a very high prevalence of contamination is observed (and the resources instead focused on addressing the cause of contamination).
• Higher sampling rates allow for compliance with more stringent regulatory limits; guidelines that coordinate sampling rates and regulatory limits would make better use of the results from PWS.
• Surveillance agencies verifying the quality of large PWS as tested by the Suppliers do not typically sample often enough to make actionable conclusions about the water quality or to challenge the Supplier's sampling results. Resources could be better spent verifying Suppliers' labs or sampling procedures.
• Regulations that require finding no samples positive for FIB provide a false sense of security to those PWS that meet them, discourage sampling beyond recommended rates, and do not provide additional sampling recommendations for agencies.

To improve the efficacy of monitoring programs, sampling recommendations should incorporate the additional factors we highlighted (contamination prevalence, desired confidence, regulatory evaluation criterion, and regulatory limit). Additionally, extending the Water Safety Plan framework (Bartram et al., 2009) to differentiate sampling recommendations by the risks identified in a PWS can also be useful; for example, PWS with low residual disinfectants should focus on improving disinfection practices instead of additional testing for FIB. Additionally, guidelines should clarify separate roles and sampling requirements for suppliers and surveillance agencies to ensure the best use of both types of agencies' resources.

Additional research is required to improve the cost-effectiveness of sampling. First, as we have highlighted throughout the manuscript, the locations and timings of sampling could be optimized. While the GDWQ recommend that verification microbial sampling should focus on locations with the “best possible chance of detecting contamination,” water suppliers and surveillance agencies could benefit greatly from clearer suggestions about how to stratify their sampling procedures throughout the stages of water system, the timing of supplies, and the geographic extent of the network. For example, if more information were available on the spatial distribution of samples, we would be able to distinguish between temporal and spatial trends; similarly, if contamination mechanisms were modeled in each PWS, sampling locations could be optimized. Second, better statistical methods to make use of the data provided by quantitative tests for FIB could be developed (e.g. our methodology treated all FIB tests as pass/fail, and therefore discarded their quantitative information). Finally, new methods could be created to combine operational monitoring parameters (such as disinfectant residual and turbidity measurements) with microbial test results, as these operational parameters are often less expensive and less time-consuming to measure.
