# Supplementary material for: Efficacy of microbial sampling recommendations and practices in sub-Saharan Africa
Source: Water Res. 2018 May 1;134:115–25. doi: 10.1016/j.watres.2018.01.054 (PMC5842043; doi:10.1016/j.watres.2018.01.054)
Supplement: Online data [file mmc1.pdf]

Supporting Information for "Efficacy of Microbial Sampling Recommendations and Practices in sub-Saharan Africa"

Contents of this file

1. Text S1
2. Table S1 and S2
3. Figures S1, S2, and S3

Introduction

This supporting information contains Text S1, which provides details on the numerical integration of beta distributions; as well as Tables S1 and S2, which detail PWS sizes and sampling rates; as well as Figures S1 and S2, which show repeat sampling and sampling rates by dataset, and Figure S3, which shows the effect of baseline water quality on the samples required to detect quality changes.

1. Text S1. Details on numerical integration of beta distributions

The paper considered how many samples would be required before a PWS could confidently conclude that an observed increase in contamination prevalence by ten percentage points was due to an actual increase in the contamination prevalence of the PWS. Given an observed contamination prevalence $\hat{p_i}$ in n_i samples, the true contamination prevalence p_i is a random variable. Since we model the sampling process as Binomially distributed, p_i follows a Beta distribution. Using the assumption that without any samples, the PWS' contamination was totally unknown (i.e. a uniform prior $\sim U[0,1]$), the Beta distribution's parameters α and β are given by Equations 1 and 2. Using $f_i(x)$ and $F_i(x)$ to represent the probability density function and the cumulative density function of p_i, the likelihood of there being in increase in the actual contamination rate ($\Pr(p_2 > p_1)$) is given by Equation 3.

$$\alpha_i(\hat{p}_i, n_i) = \hat{p}_i n_i + 1 \tag{1}$$

$$\beta_i(\hat{p}_i, n_i) = (1 - \hat{p}_i) n_i + 1 \tag{2}$$

$$\Pr(p_2 > p_1) = \int_0^1 f_2(x; \alpha_2, \beta_2) F_1(x; \alpha_1, \beta_1)) dx \tag{3}$$

To determine the number of samples required to detect a performance degradation with confidence level c, given the observation of an increase in $\hat{p}$ by 10 percentage points (0.1), Equation 4 was solved numerically for the minimum required number of samples N. The minimum time interval for quality comparisons was then equal to N divided by the mean sampling frequency.

$$\begin{aligned} &\min_{N} \\ &\text{s.t.} \quad \int_0^1 f_2(x; \alpha(\hat{p_1} + 0.1, N), \beta(\hat{p_1} + 0.1, N)) F_1(x; \alpha(\hat{p_1}, N), \beta(\hat{p_1}, N)) dx \geq c \end{aligned} \tag{4}$$

Table S1: Number of PWS serving populations of different sizes, split by country. Population data was not included for Ghana, Guinea, or Senegal.

Country	<5,000	5,000-100,000	100,001-500,000	>500,000	N/A[a]	Total
Benin	4	4	1	0	2	11
Ethiopia	0	2	1	0	1	4
Ghana	0	0	0	0	3[b]	3
Guinea	0	0	0	0	39[b]	39
Kenya	0	1	7	1	0	9
Senegal	0	0	0	0	43[b]	43
Uganda	122	56	8	0	9	195
Zambia	10	11	3	0	23	47
Total	136	74	20	1	120	351

[a] Population data not available
[b] We were unable to obtain relevant population data for the towns and cities in the dataset from Ghana, Guinea, or Senegal

Table S2: Observed annual sampling rates for PWS, disaggregated by country.

Country	Mean	Median	Range
Benin	134	72	(21, 700)
Ethiopia	1912	232	(15, 7168)
Ghana	222	222	(163, 282)
Guinea	71	12	(1, 1240)
Kenya	442	54	(1, 3491)
Senegal	13	12	(0[a], 39)
Uganda	29	20	(0[a], 350)
Zambia	80	34	(0[a], 1027)
Total	76	12	(0[a], 7168)

[a] A zero indicates a PWS in the MfSW-database that conducted all of its water quality tests outside of the one-year observation window in which annual rates were calculated.

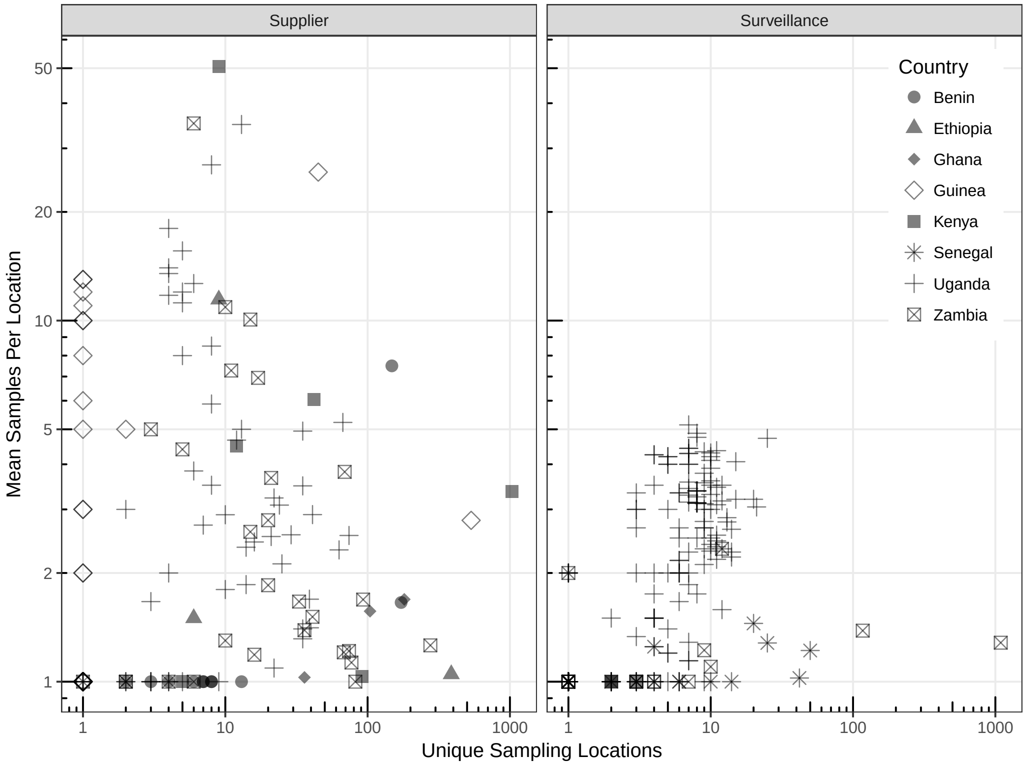

Figure S1: The number of unique sampling locations and the mean number of samples per location for each PWS in the dataset, separated by country. Samples without location information are excluded. Since the same location may be described differently by different sampling agents, the unique locations is an overestimate and the samples per location is an underestimate.

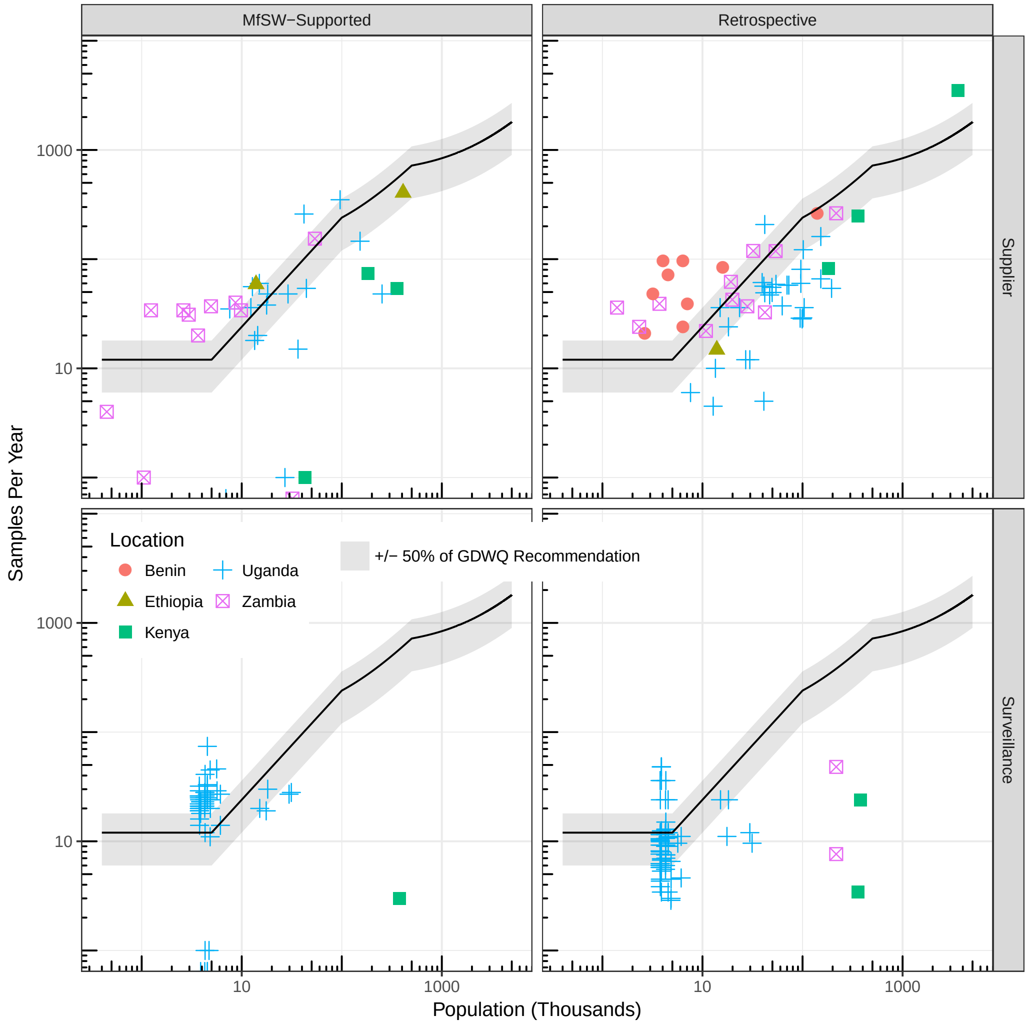

Figure S2: MfSW's Effect on Sampling Rates. Annual equivalent sampling rates by institutions compared with GDWQ Recommendation (black line) and +/- 50% of GDWQ Recommendation (shaded gray) by supplier and surveillance agencies under MfSW-supported data collection activities and using retrospective data. Each PWS in the MfSW-supported and retrospective datasets is shown as a separate dot, with Benin (red dots), Ethiopia (brown triangles), Kenya (green squares), Uganda (blue crosses) and Zambia (pink crossed boxes)

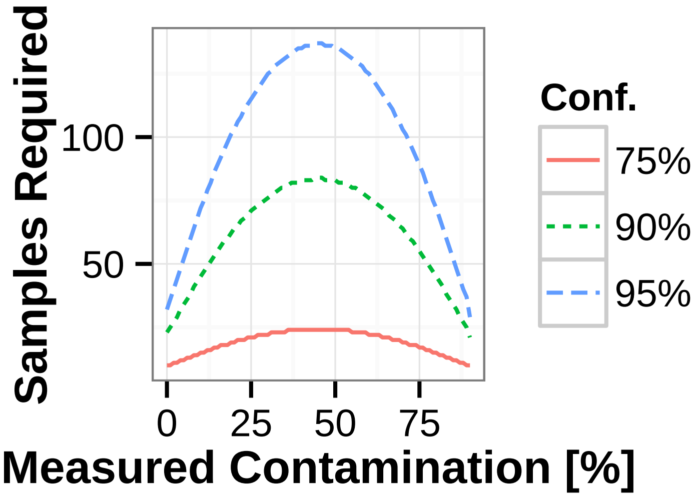

Figure S3: The samples required to detect an increase in contamination by ten percentage points depends on the required confidence level and the initially observed contamination rate. The variance and therefore the uncertainty of a binomial Bernoulli process is highest when the probability of success is 50%. An adaptive sampling requirement would impose more frequent sampling requirements on PWS with contamination rates closer to 50%. The peak here is below 50% because we considered the ability to detect a 10% increase in contamination.
